# Projected impact, cost-effectiveness, and budget implications of rotavirus vaccination in Mongolia

**DOI:** 10.1016/j.vaccine.2018.12.056

**Published:** 2019-02-04

**Authors:** Munkh-Erdene Lusvan, Frédéric Debellut, Andrew Clark, Sodbayar Demberelsuren, Dashpagam Otgonbayar, Tselkhaasuren Batjargal, Sugarmaa Purevsuren, Devin Groman, Jacqueline Tate, Clint Pecenka

**Affiliations:** a School of Public Health, Mongolian National University of Medical Science, Rm. 334, Sukhbaatar District, S. Zorig Street, Ulaanbaatar, Mongolia; b PATH, Rue de Varembé 7, 1202 Geneva, Switzerland; c London School of Hygiene and Tropical Medicine, Keppel Street, London WC1E 7HT, United Kingdom; d World Health Organization Representative Office Mongolia, Government Building VIII, Olympic Street 2, Sukhbaatar District, Ulaanbaatar 14210, Mongolia; e National Center for Communicable Disease, Ministry of Health, Government Building VIII, Olympic Street 2, Sukhbaatar District, Ulaanbaatar 14210, Mongolia; f PATH, 2201 Westlake Ave, Suite 200, Seattle, WA 98121, USA; g Centers for Disease Control and Prevention, Atlanta, GA, USA

**Keywords:** Rotavirus, Vaccination, Cost-effectiveness, DALY, ICER, Mongolia

## Abstract

**Introduction:**

Rotavirus disease in Mongolia is estimated to cause more than 50 deaths yearly and many more cases and hospitalizations. Mongolia must self-finance new vaccines and does not automatically access Gavi prices for vaccines. Given the country’s limited resources for health, it is critical to assess potential new vaccine programs. This evaluation estimates the impact, cost-effectiveness, and budget implications associated with a nationwide rotavirus vaccine introduction targeting infants as part of the national immunization program in Mongolia, in order to inform decision-making around introduction.

**Methods:**

The analysis examines the use of the two-dose vaccine ROTARIX®, and three-dose vaccines ROTAVAC® and RotaTeq® compared to no vaccination from the government and the societal perspective. We use a modelling approach informed by local data and published literature to analyze the impact and cost-effectiveness of rotavirus vaccination over a ten-year time period starting in 2019, using a 3% discount rate. Our main outcome measure is the incremental cost-effectiveness ratio (ICER) expressed as US dollar per DALY averted. We assessed uncertainty around a series of parameters through univariate sensitivity analysis.

**Results:**

Rotavirus vaccination in Mongolia could avert more than 95,000 rotavirus cases and 271 deaths, over 10 years. Averted visits and hospitalizations represent US$2.4 million in health care costs saved by the government. The vaccination program cost ranges from $6 to $11 million depending on vaccine choice. From the governmental perspective, ICER ranged from $412 to $1050 and from $77 to $715 when considering the societal perspective. Sensitivity analysis highlights vaccine price as the main driver of uncertainty.

**Conclusion:**

Introduction of rotavirus vaccination is likely to be highly cost-effective in Mongolia, with ICERs estimated at only a fraction of Mongolia’s per capita GDP. From an economic standpoint, ROTAVAC® is the least costly and most cost-effective product choice.

## 1 Introduction

Child mortality has been declining worldwide as a result of socioeconomic development and child survival interventions. However, diarrhea remains a primary cause of mortality, and rotavirus is responsible for a large share of these deaths. In 2013, rotavirus was responsible for 215,000 of the 578,000 childhood diarrheal deaths worldwide with more than 90% of these deaths occurring in low- and middle-income countries [1]. In Mongolia, the World Health Organization (WHO) and the Centers for Disease Control and Prevention estimated that over 50 children under five years of age die of rotavirus infection annually. Diarrhea is also a leading cause of childhood morbidity in Mongolia [2], [3], [4], [5], [6]. A 2013 social indicator sample survey reported a prevalence of diarrhea of 8.2% in children under five years, with higher prevalence in rural areas. The highest period-prevalence is seen among children 12–23 months [7].

Since 2009, WHO has recommended rotavirus vaccines to be included in national immunization programs as part of a comprehensive strategy to control childhood diarrheal diseases [8]. Two rotavirus vaccines have been licensed and used globally: the monovalent vaccine ROTARIX® (GlaxoSmithKline Biologicals, Rixensart, Belgium) and the pentavalent vaccine RotaTeq® (Merck & Co, Inc., West Point, PA, USA). Clinical trials and post-introduction evaluations have indicated vaccine efficacy and/or effectiveness ranges from approximately 70% to 100% in high- and upper-middle-income countries to approximately 50% to 70% in lower-income countries in Africa and Asia [9], [10]. ROTAVAC® (Bharat Biotech International Limited, India), another monovalent vaccine, received WHO prequalification for use globally in early 2018 and has been licensed and used in India since 2017 [11]. Data from a clinical trial in India, a lower-middle-income setting where efficacy is expected to be lower, showed 56% efficacy of ROTAVAC® [12].

One of the objectives of Mongolia’s National Program on Infectious Diseases Prevention and Control is to use surveillance-based evidence to inform decision-making around new vaccine introduction. Since 2009, Mongolia has participated in the WHO Western Pacific Regional Office rotavirus surveillance network to collect data on the rotavirus-positive proportion of diarrhea cases admitted to hospitals. In addition, Mongolia’s comprehensive multi-year plan recommends that rotavirus vaccines be considered for the routine childhood immunization schedule in the near future [13]. Mongolia recently transitioned from Gavi support and has to fully self-finance any new vaccines. As a transitioned country, Mongolia cannot automatically access Gavi-negotiated prices for ROTARIX®, the most common product choice among Gavi countries. In this situation, impact, cost-effectiveness, affordability, and budget impact are among the key factors to inform decisions around introducing rotavirus vaccination and which product to choose. The purpose of this evaluation is to estimate the potential impact, cost-effectiveness, and budget implications associated with infant rotavirus vaccination as part of the national immunization program in Mongolia compared to no vaccination, assessing the three different rotavirus products available at the time of writing, and to provide data needed to prepare for potential introduction.

## 2 Materials and methods

The immunization program in Mongolia has been very successful at maintaining very high coverage for traditional antigens. While the Ministry of Health is responsible for policy making and setting health regulations, immunization services are part of the health services provided by local governments and city corporations in district health facilities and family health centers [13].

This analysis examines infant vaccination with ROTARIX® administered at 2 and 4 months along with Diphtheria, Tetanus, and Pertussis (DTP) at DTP dose 1 (DTP1) and DTP dose 2 (DTP2). We also examine ROTAVAC® and RotaTeq® vaccines administered to the same group, following the same schedule, with a third dose at 6 months along with DTP dose 3 (DTP3). We compare the impact and cost-effectiveness of rotavirus vaccination to no vaccination, over a ten-year time period starting in 2019. Our analysis viewed 2019 as a potential timeline for starting a rotavirus vaccination program if it became a priority based on other ongoing immunization activities and looks at ten years to inform medium term planning and budgeting.

**Fig. 1:**
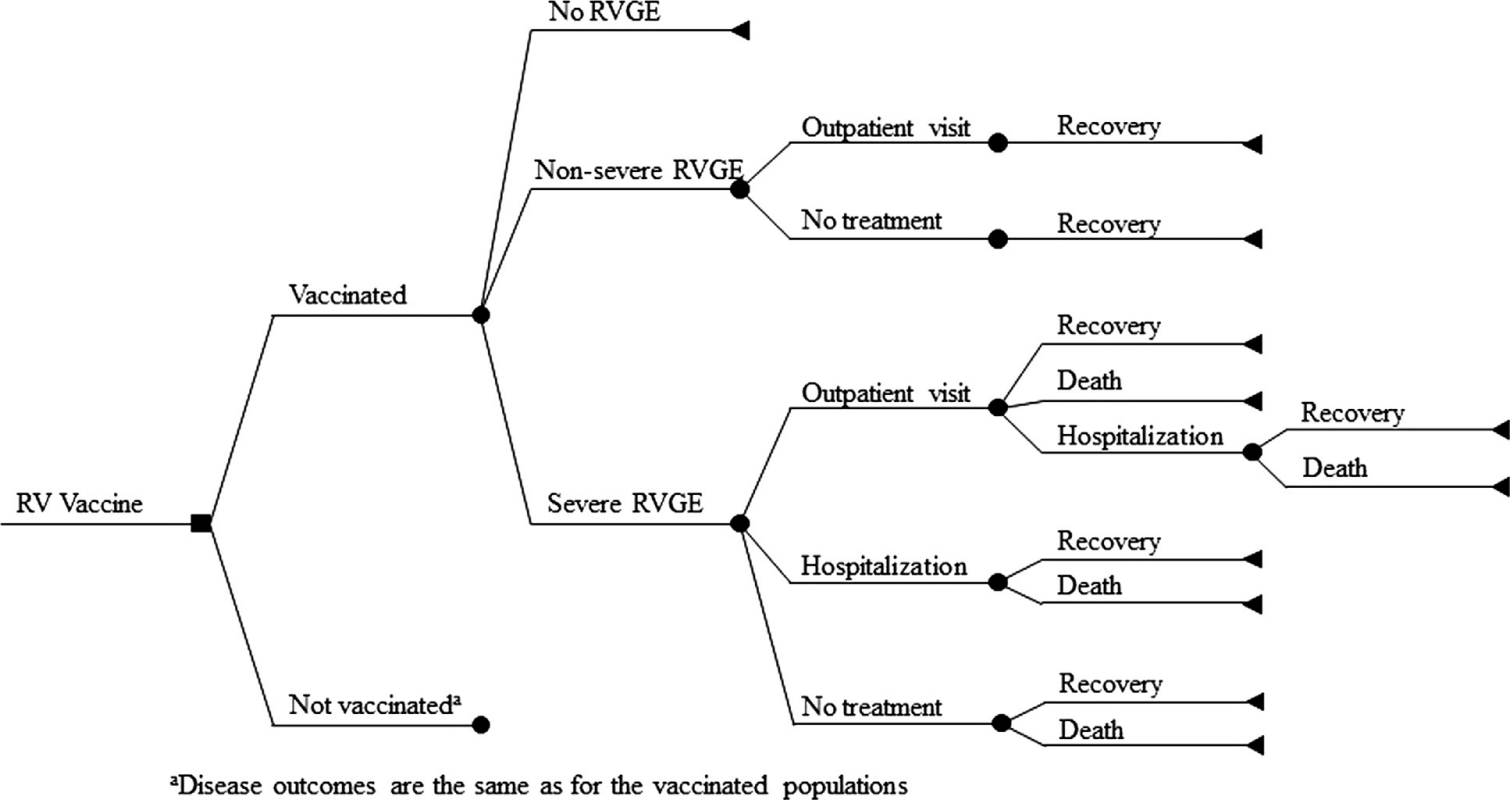
Model schematic.

When infected with rotavirus, children may or may not get rotavirus gastroenteritis (RVGE). Children with RVGE will experience a non-severe or severe episode. Non-severe episodes result in recovery with or without outpatient care. Severe episodes result in recovery or death with or without outpatient and/or inpatient care (Fig. 1). This analysis tracks costs and benefits of rotavirus disease and rotavirus vaccination of infants over 10 consecutive birth cohorts following each cohort until they reach age 5. We exclude any indirect benefits of vaccination, assuming herd effect would have minimal impact considering the high vaccine coverage in Mongolia. Costs and benefits are discounted using a 3% annual rate [14]. Results are presented from the government and the societal perspective. All monetary units were adjusted to 2017 US$ using currency exchange rate from the Central Bank of Mongolia (US$ 1 = MNT 2438) and the U.S. Consumer Price Index (CPI) from the United States Department of Labor, Bureau of Labor Statistics.

We calculate the incremental cost-effectiveness ratio (ICER) by dividing the difference between incremental vaccine program costs and averted health care costs by the number of disability-adjusted life-years (DALY) averted by the intervention. We elected to use the discounted cost per averted DALY as our main outcome measure. DALYs are commonly use in cost-effectiveness analysis, partly because of their comparability across settings and interventions. Additional outcomes measured include the number of deaths, hospitalizations, outpatient visits, and cases averted; the incremental cost of the vaccination program; and health care costs averted. This analysis examines budget implications, providing undiscounted annual costs of the vaccination program over the ten-year time horizon of the analysis.

### 2.1 Model

We used UNIVAC (version 1.3.07), a universal static impact and cost-effectiveness decision support model used in many studies worldwide [15], [16], [17], [18]. Developed for use in low- and middle-income countries, UNIVAC provides estimates of the potential impact and cost-effectiveness of rotavirus and other vaccines. UNIVAC follows methods developed for the TRIVAC model, which have been described elsewhere [18]. Model inputs include burden of disease, vaccine schedule, efficacy, coverage, timeliness of vaccination, vaccine program costs, and health care costs. Input parameters were collected from the literature and local sources to reflect country context. All data inputs and assumptions were discussed and validated by a group of experts that participated in this cost-effectiveness analysis. The group of experts included members from the Expanded Programme on Immunization and the National Center for Communicable Diseases; staff from the Sukhbaatar district surveillance hospital; the surveillance department of the National Maternal and Child Disease Center; the National Center for Health Development; and the WHO.

### 2.2 Disease burden

We estimated severe and non-severe RVGE cases, outpatient visits, and hospitalizations in Mongolia using data from different sources. In the absence of RVGE incidence data, we based our estimates on health facility data and then adjusted for treatment seeking to estimate RVGE incidence. We first calculated rates of outpatient visits and hospitalizations using the number of diarrhea cases in children under five years (outpatient and inpatient) reported by the Center for Health Development and population data from the National Statistical Office [19], [20]. We assumed all hospitalizations were for severe diarrhea and applied the rotavirus-attributable fraction observed among diarrhea admissions in two hospitals of Ulaanbaatar city reported by the surveillance network in 2015 [21]. As rotavirus positivity is usually lower in outpatient settings, we applied half the rotavirus-attributable fraction to determine non-severe RVGE relative to all diarrhea [22]. After obtaining estimates of health facility utilization, we then calculated incidence of RVGE as 4729 per 100,000 children under five years, using a treatment seeking rate of 46.8% for outpatient visits [7] and a rate of 80% for hospitalizations, assuming that a larger proportion of severe cases seek treatment in an inpatient setting. Local information suggests that children often visit an outpatient setting before being hospitalized, resulting in facility utilization that exceeds the number of severe cases. To account for uncertainty, we examined other potential incidence rates. As a high input, we used 10,000 per 100,000 cases in children under five years as demonstrated by Bilcke et al. in a global systematic review and meta-analysis [23], which is consistent with the incidence level observed by Zaman et al. in the placebo arm of the RotaTeq® randomized controlled trial in Bangladesh and Vietnam [24]. As a low input, we used our base case assumptions accounting for rotavirus positivity observed in other countries of the region [25], [26]. We used a disease mortality rate of 18 per 100,000 (16.5–20) per year in children under five years as reported by Tate et al. [1]. Disease event age distribution is based on surveillance data [21]. DALY weights for moderate and severe diarrhea in 2013 were 0.188 and 0.247, respectively [27]. We assumed the average duration of rotavirus disease to be 3 days for non-severe cases and 7 days for severe cases [28]. All data inputs used to characterize disease burden are displayed in Table 1.

**Table 1:** Input parameters for estimating disease burden.

Parameter	Estimate	Scenarios		Source/s
		Low	High	
*Annual incidence per 100,000 < 5 years*				
Rotavirus (non-severe) cases	3060	–	–	Assumption derived from [7], [19], [20], [21]
Rotavirus (non-severe) outpatient visits	1432	–	–	Assumption derived from [7], [19], [20], [21]
Rotavirus (severe) cases	1670	–	–	Assumption derived from [7], [19], [20], [21]
Rotavirus (severe) outpatient visits	781	–	–	Assumption derived from [7], [19], [20], [21]
Rotavirus (severe) hospitalizations	1336	795	2825	Assumption derived from [7], [19], [20], [21]
Rotavirus deaths	18	16.5	20	[1]

*Disability weight for DALY calculations*				
Rotavirus (non-severe) cases	18.8%	–	–	[27]
Rotavirus (severe) cases	24.7%	–	–	[27]

*Mean duration of illness (in days)*				
Rotavirus (non-severe) cases	3	–	–	[28]
Rotavirus (severe) cases	7	–	–	[28]

*Cumulative age distribution of disease, cases, and deaths*				
<1 m:	4%	–	–	Assumption
<2 m:	7%	–	–	Assumption
<3 m	11%	–	–	Assumption
<6 m:	22%	–	–	[21]
<12 m:	70%	–	–	[21]
<24 m:	97%	–	–	[21]
<36 m:	98%	–	–	Assumption
<48 m:	99%	–	–	Assumption
<59 m:	100%	–	–	[21]

### 2.3 Vaccine coverage and efficacy

As rotavirus vaccine is anticipated to be delivered alongside DTP according to WHO recommendations, we use DTP coverage as reported by WHO and UNICEF, assuming constant coverage across the study period [29]. Coverage for DTP2 was assumed to be halfway between DTP1 and DTP3. We assumed that timeliness of rotavirus vaccination would be similar to that for DTP reported in the 2013 Mongolia Multiple Indicator Cluster Survey (MICS) study [30]. Efficacy and waning inputs are based on work from Clark et al., who estimated efficacy of rotavirus vaccination by time since administration of the last dose, extracting data from all randomized controlled trials in very low-, low-, and high-mortality settings [31]. Mongolia data inputs correspond to low child mortality and 81% (62% − 87%) efficacy two weeks after receiving the last dose. We further assumed that one dose of vaccine would confer half the efficacy of the last dose. In the absence of data to support different efficacy for different rotavirus vaccines, we assume similar efficacy and waning level for ROTARIX®, RotaTeq® and ROTAVAC®, as done in similar analyses [32]. Per model input requirements, the same efficacy value is applied to all endpoints. Input parameters for vaccine coverage, timeliness, and vaccine impact are available from Table 2.

**Table 2:** Input parameters for estimating rotavirus vaccine (RV) coverage, timeliness and health impact.

Parameter	Estimate	Scenarios		Source/s
		Low	High	
**Rotavirus vaccine (RV) coverage and timeliness parameters**				
*Total coverage in year 2019*				
DTP1	99%	70%	100%	[29]
DTP2	99%	70%	100%	[29]
DTP3	99%	70%	100%	[29]

*Percent of total coverage of DTP1 achieved by age in year 2019 (proxy for RV doses given with DTP1)*				
6 m	94%	70%	100%	[7], [30]
12 m	95%	70%	100%	
24 m	95%	70%	100%	

*Percent of total coverage of DTP2 achieved by age in year 2019 (proxy for RV doses given with DTP2)*				
6 m	91%	64%	92%	[7], [30]
12 m	92%	70%	100%	
24 m	92%	70%	100%	

*Percent of total coverage of DTP3 achieved by age in year 2019 (proxy for RV doses given with DTP3)*				
6 m	86%	61%	87%	[7], [30]
12 m	89%	70%	100%	
24 m	89%	70%	100%	

**Rotavirus vaccine (RV) health impact parameters**				
*Vaccine efficacy 2 weeks after vaccination*				
1 dose	41%	31%	44%	Assumption
2 doses	81%	62%	87%	[31]
3 doses	81%	62%	87%	[31]

*Duration of vaccine efficacy, 1 dose*				
After 6 m	36%	28%	39%	[31]
After 12 m	36%	27%	38%	[31]
After 24 m	34%	26%	37%	[31]

*Duration of vaccine efficacy, 2 doses*				
After 6 m	72%	55%	78%	[31]
After 12 m	71%	54%	76%	[31]
After 24 m	68%	52%	73%	[31]

*Duration of vaccine efficacy, 3 doses*				
After 6 m	72%	55%	78%	[31]
After 12 m	71%	54%	76%	[31]
After 24 m	68%	52%	73%	[31]

### 2.4 Vaccine price and delivery cost

Mongolia transitioned out of support from Gavi, the Vaccine Alliance in 2016 and now fully self-finances new vaccines [33]. Although Gavi-secured pricing agreements with manufacturers allow fully self-financing countries to benefit from Gavi-like prices for some vaccines [34], it does not automatically apply to Mongolia for use of ROTARIX® as the country did not adopt while still eligible for support. To introduce ROTARIX®, the Mongolian government will have to enter into negotiations with the manufacturer [35], [36]. As the potential outcome of this negotiation is uncertain, we used price data reported by other middle-income countries that do not access Gavi prices [37]. The average 2016 price was $6.20 per dose [38]. We utilized prices of $1.00 and $3.50 per dose for ROTAVAC® and RotaTeq®, respectively [34], [39].

In addition to vaccine price, we included procurement of safe disposal bags at a unit price of $0.80. We assume a standard wastage rate of 5% for ROTARIX® and RotaTeq® and 25% for ROTAVAC®, based on the number of doses per container. While ROTARIX® and RotaTeq® have a single dose presentation, ROTAVAC® is available in 10- or 5-dose vial, potentially generating higher wastage [11], [40]. We assume international handling and delivery of vaccines will represent 3.5% and 8% of vaccine price respectively [41], [42].

To our knowledge, no costing study has been carried out in Mongolia to inform the incremental health system cost of delivering rotavirus vaccine. We use data from a 2018 systematic review on the cost of immunization programs, using incremental cost-per-dose estimates reported by middle-income countries [43]. Data inputs used to estimate the vaccine program cost are available from Table 3.

**Table 3:** Input parameters for estimating health service costs and rotavirus vaccine program costs (2017 US$).

Parameter	Estimate	Scenarios		Source/s
		Low	High	
*Rotavirus program costs*				
International handling (% of vaccine price)	3.5%	–	–	Assumption
International delivery (% of vaccine price)	8%	–	–	Assumption
Incremental system cost per dose	$1.91	$0.50	$2.50	[43], assumption for Low and High
ROTARIX® vaccine price per dose	$6.20	$2.02	$15.76	[38]
ROTARIX® wastage	5%	–	–	Assumption
RotaTeq® vaccine price per dose	$3.50	$3.20	$7	[11], assumption for Low and High
RotaTeq® wastage	5%	–	–	Assumption
ROTAVAC® price per dose	$1	$0.5	$2	[11], assumption for Low and High
ROTAVAC® wastage	25%	–	–	Assumption

*Health service costs*				
Government cost per outpatient visit	$7.29	$3.65	$14.58	Modelled using WHO-CHOICE, assumption for Low and High
Household cost per outpatient visit	$2.64	$1.32	$5.28	
Government cost per inpatient admission	$77.93	$38.97	$155.86	
Household cost per inpatient admission	$96.27	$48.13	$192.54	

### 2.5 Health service costs

Outpatient cost estimates were based on WHO-CHOICE service delivery unit cost estimates for Mongolia as well as commodity costs. We assumed government cost encompasses direct medical cost, while households cost includes direct non-medical costs. We did not include indirect costs, a conservative assumption due to lack of data. Cost per outpatient visit is the sum of the WHO-CHOICE cost in a primary facility setting and 6 oral rehydration salts (ORS) packets per day for 3 days, valuing ORS solution at $0.29 per packet, per MSH’s International Medical Products Price Guide [44]. Based on a 2017 PATH literature review examining all diarrhea cost-of-illness studies reporting data for low- and middle-income countries, we calculated that direct medical costs accounted for 70% of all direct costs. We applied this percentage to calculate direct non-medical costs. We estimated the government cost of an outpatient visit at $7.29 and households cost at $2.64 (Table 3).

Inpatient costs were estimated using a mix of local data available from an unpublished cost of rotavirus diarrhea study and modelled data based on 2010 WHO-CHOICE data. The study estimated household costs linked to an episode of diarrhea, accounting for households’ preparation and transportation costs, and out-of-pocket payments prior to and during hospitalization. The study also collected information from medical records to assess hospital direct costs. This information was combined with WHO-CHOICE data on bed day costs at a secondary level hospital for Mongolia and a four-day length of stay. We estimated the government cost of an inpatient stay at $77.93 and the households cost at $96.27 (Table 3).

### 2.6 Scenario analysis and sensitivity analysis

We examined scenarios assessing the use of other available rotavirus vaccines RotaTeq® and ROTAVAC® and also used univariate sensitivity analysis to highlight the main drivers of cost-effectiveness of rotavirus vaccination in Mongolia. Parameters explored in the sensitivity analysis include incidence of severe RVGE hospitalizations, incidence of RVGE deaths, vaccine efficacy, vaccine coverage, vaccine price per dose, incremental health system cost per dose, inpatient admission costs, outpatient visit costs, and discount rate. All parameters range values used for the sensitivity analysis are available from Table 1, Table 2, Table 3.

## 3 Results

### 3.1 Base case scenario

Between 2019 and 2028, a rotavirus vaccination program in Mongolia has the potential to prevent more than 62,000 non-severe RVGE cases, approximately 34,000 severe cases, and 271 deaths. This corresponds to approximately 19,000 undiscounted DALYs averted. We estimate that rotavirus vaccination would avert 44,900 outpatient visits and more than 27,000 hospitalizations, representing $2.4 and $5 million in undiscounted health care costs saved from the government and societal perspectives, respectively. The use of ROTARIX® would result in a cost-effectiveness ratio of $1050 per DALY averted from the government perspective and $715 from the societal perspective. Additional details on health and economic benefits of rotavirus vaccination are available from Table 4.

**Table 4:** Health and economics benefits (10 cohorts vaccinated over the period 2019–2028, costs discounted, 2017 US$).

ROTARIX®			
	No vaccine	With vaccine	Averted
Total non-severe cases <5 yrs	93,624	31,530	62,095
Total severe cases <5 yrs	51,096	17,207	33,888
Total outpatient visits	67,709	22,802	44,907
Total hospitalizations	40,877	13,766	27,111
Total deaths <5 yrs	409	138	271

DALYs	10,701	3607	7094
*Total government health service costs*			
Total outpatient visit costs	$430,306	$145,031	$285,275
Total hospitalization costs	$2,777,024	$935,974	$1,841,050

*Total societal health service costs*			
Total outpatient visit costs	$586,137	$197,553	$388,584
Total hospitalization costs	$6,207,592	$2,092,220	$4,115,372

*Total vaccination program costs*	–	$9,574,121	–

RotaTeq®			

	No vaccine	With vaccine	Averted

Total non-severe cases <5 yrs	93,624	28,815	64,809
Total severe cases <5 yrs	51,096	15,726	35,370
Total outpatient visits	67,709	20,839	46,870
Total hospitalizations	40,877	12,581	28,296
Total deaths <5 yrs	409	126	283
DALYs	10,701	3293	7408

*Total government health service costs*			
Total outpatient visit costs	$430,306	$132,435	$297,871
Total hospitalization costs	2,777,024	854,684	1,922,341

*Total societal health service costs*			
Total outpatient visit costs	586,137	180,395	405,742
Total hospitalization costs	6,207,592	1,910,508	4,297,084

*Total vaccination program costs*	–	$9,407,658	–

ROTAVAC®			

	No vaccine	With vaccine	Averted

Total non-severe cases <5 yrs	93,624	28,815	64,809
Total severe cases <5 yrs	51,096	15,726	35,370
Total outpatient visits	67,709	20,839	46,870
Total hospitalizations	40,877	12,581	28,296
Total deaths <5 yrs	409	126	283
DALYs	10,701	3293	7408

*Total government health service costs*			
Total outpatient visit costs	$430,306	$132,435	$297,871
Total hospitalization costs	2,777,024	854,684	1,922,341

*Total societal health service costs*			
Total outpatient visit costs	586,137	180,395	405,742
Total hospitalization costs	6,207,592	1,910,508	4,297,084

*Total vaccination program costs*	–	$5,274,441	–

**Fig. 2:**
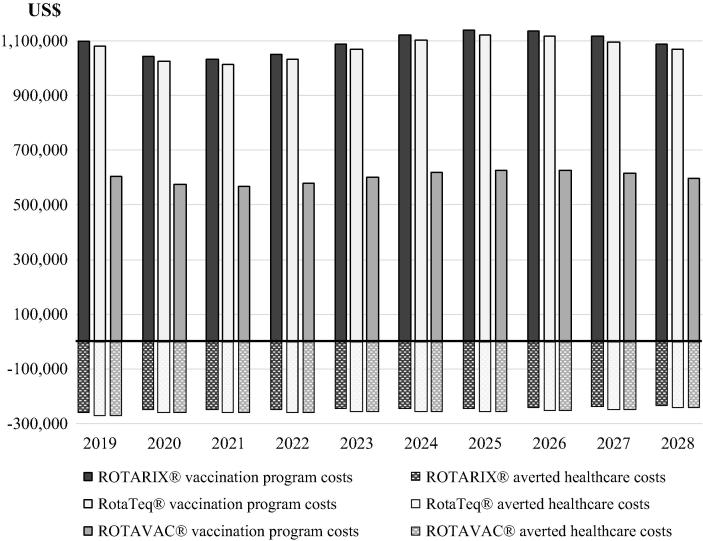
Budget implications (Government perspective, all figures undiscounted).

The cost of the vaccination program is projected to average $1.1 million per year, with the majority of the cost dedicated to vaccine procurement (79%). This amount would be partially balanced by savings made on health care costs averted, but with ROTARIX® at a price per dose of $6.20, the rotavirus vaccination program would still represent a net cost of about $850,000 on average per year. Fig. 2 shows the anticipated base case budget implications by year.

### 3.2 Additional scenarios and one-way sensitivity analyses

**Fig. 3:**
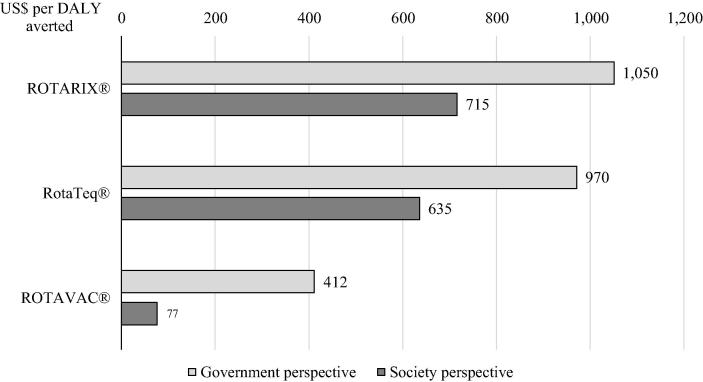
Scenario analysis results: cost per DALY averted in US$ (costs and benefits discounted).

We also explored the use of RotaTeq® and ROTAVAC® vaccines instead of ROTARIX®. Fig. 3 presents the cost per DALY averted for each scenario and perspective. From the societal perspective, the cost per DALY is $77 with ROTAVAC® and $635 with RotaTeq®. From the government perspective, the cost per DALY averted is $412 with ROTAVAC® and $970 with RotaTeq®.

The undiscounted results from the government perspective (Table 5) indicate that budget impact varies greatly depending on which vaccine is considered. While ROTARIX® and RotaTeq® represent a total vaccination program cost of almost $11 million, ROTAVAC® results in a much lower cost of about $6 million. Accounting for savings on health care costs averted from the government perspective, the net cost of the vaccination program with ROTAVAC® would be less than $3.5 million over 10 years.

**Table 5:** Budget impact analysis (Government perspective, all figures in undiscounted US$).

ROTARIX® ($6.30 per dose)							
	Fully vaccinated children	Health system cost	Vaccine cost	Total vaccination program	Averted Outpatient visits cost	Averted Hospitalizations cost	Net Cost
2019	59,725	228,305	871,493	1,099,798	34,645	223,584	841,569
2020	56,711	216,778	827,495	1,044,274	33,369	215,350	795,554
2021	56,072	214,344	818,204	1,032,548	33,323	215,052	784,174
2022	57,091	218,238	833,067	1,051,305	33,038	213,214	805,053
2023	59,070	225,799	861,928	1,087,727	32,862	212,078	842,787
2024	60,936	232,928	889,144	1,122,072	32,744	211,320	878,008
2025	61,895	236,592	903,130	1,139,722	32,627	210,564	896,530
2026	61,646	235,641	899,497	1,135,138	32,094	207,124	895,919
2027	60,575	231,545	883,863	1,115,408	31,603	203,952	879,854
2028	59,016	225,586	861,115	1,086,701	31,067	200,495	855,139

	592,737	2,265,757	8,648,936	10,914,693	327,373	2,112,733	8,474,587

RotaTeq® ($3.50 per dose)							

	Fully vaccinated children	Health system cost	Vaccine cost	Total vaccination program	Averted Outpatient visits cost	Averted Hospitalizations cost	Net Cost

2019	59,759	342,450	738,202	1,080,652	36,159	233,358	811,134
2020	56,743	325,165	700,941	1,026,106	34,828	224,765	766,513
2021	56,104	321,507	693,055	1,014,562	34,780	224,454	755,329
2022	57,124	327,350	705,650	1,032,999	34,482	222,535	775,982
2023	59,104	338,695	730,106	1,068,800	34,299	221,350	813,152
2024	60,971	349,393	753,168	1,102,560	34,176	220,558	847,827
2025	61,930	354,890	765,017	1,119,906	34,054	219,770	866,083
2026	61,681	353,465	761,947	1,115,412	33,497	216,179	865,735
2027	60,609	347,322	748,703	1,096,025	32,984	212,868	850,173
2028	59,049	338,382	729,432	1,067,814	32,425	209,260	826,128

	593,073	3,398,618	7,326,219	10,724,837	341,685	2,205,096	8,178,056

ROTAVAC® ($1 per dose)							

	Fully vaccinated children	Health system cost	Vaccine cost	Total vaccination program	Averted Outpatient visits cost	Averted Hospitalizations cost	Net Cost

2019	59,759	342,450	263,422	605,872	36,159	233,358	336,354
2020	56,743	325,165	250,125	575,290	34,828	224,764	315,698
2021	56,104	321,507	247,311	568,818	34,780	224,453	309,585
2022	57,124	327,350	251,806	579,155	34,482	222,535	322,138
2023	59,104	338,695	260,533	599,227	34,299	221,350	343,579
2024	60,971	349,393	268,762	618,155	34,176	220,558	363,421
2025	61,930	354,890	272,990	627,880	34,054	219,769	374,057
2026	61,681	353,465	271,895	625,360	33,497	216,179	375,684
2027	60,609	347,322	267,169	614,491	32,984	212,868	368,639
2028	59,049	338,382	260,292	598,674	32,425	209,260	356,989

	593,073	3,398,618	2,614,305	6,012,923	341,684	2,205,094	3,466,145

**Fig. 4:**
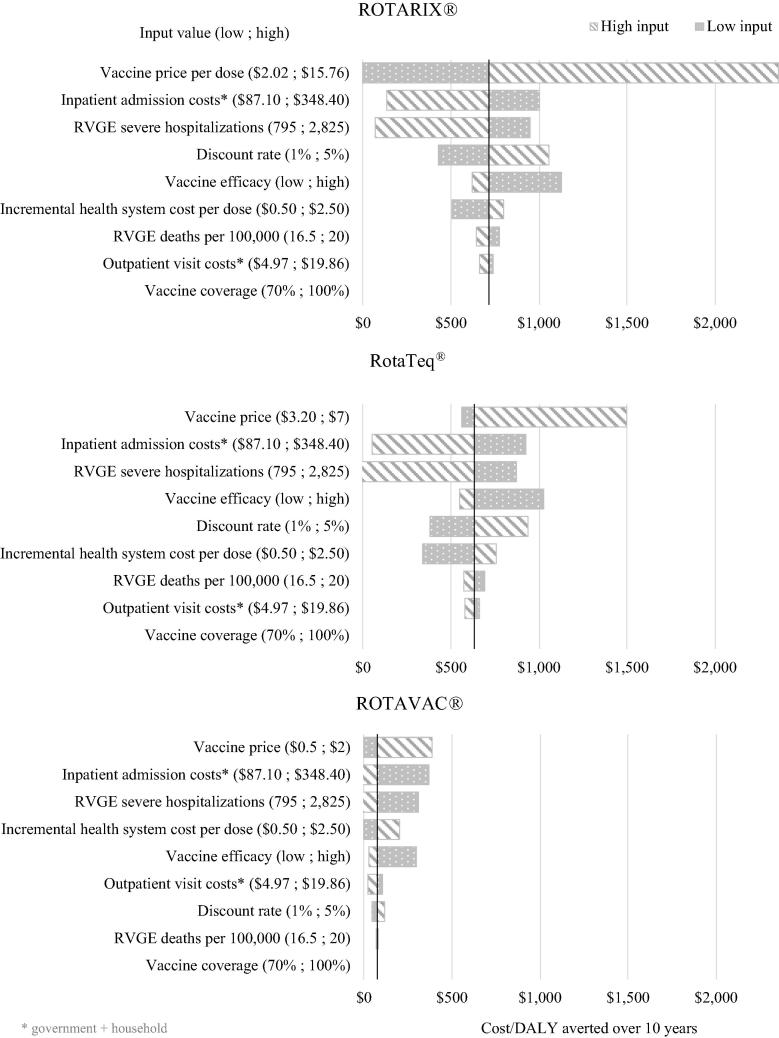
One-way sensitivity analysis of cost per DALY averted over 10 years (societal perspective).

Results of the one-way sensitivity analysis show that vaccine price is a key driver of uncertainty of the results (Fig. 4). Other drivers of uncertainty include the inpatient admission costs and the rate of severe RVGE hospitalizations. These drivers were consistent among products. Vaccine coverage has no impact on the ICER as it affects costs and benefits equally.

## 4 Discussion

Our analysis shows that rotavirus vaccination in Mongolia has the potential to avert a substantial disease burden and be highly cost-effective. The cost per DALY averted by rotavirus vaccination represents 28% and 19% of the GDP per capita ($3694 in 2016) from the government and the societal perspectives, respectively [45]. WHO no longer recommend to compare the ICER to GDP per capita and Mongolia has not yet defined a country specific threshold that we could use. In an attempt to allow for comparability, we elected to compare ICER to GDP per capita as done previously in the country [46]. While study results should be interpreted carefully, we believe that they present conservative estimates as we only explore the direct benefits of rotavirus vaccination. A prior cost-effectiveness study found that pneumococcal conjugate vaccination at the Gavi price would be highly cost-effective in Mongolia, similar to our findings for rotavirus vaccination [46]. In addition, many similar studies in middle-income settings found rotavirus vaccination to be a cost-effective intervention with ICERs ranging from cost saving to cost-effective [47].

While the range of incremental cost-effectiveness ratio (ICER) values shows that rotavirus vaccination is likely to be a highly cost-effective intervention at most thresholds, other critical considerations around affordability, feasibility, and status of other new vaccine introductions should be part of decision-making [48].

Budget impact varies depending on which vaccine is considered. While ROTARIX® and RotaTeq® are relatively similar from a cost perspective, there is an economic argument for Mongolia to choose ROTAVAC® for a potential introduction. With ROTAVAC®, the vaccination program cost would likely be the lowest and the most affordable with a net cost approximately 70% lower than ROTARIX® and RotaTeq®. To our knowledge, this is the first analysis that demonstrates such a clear economic argument for ROTAVAC®. While product choice is a multifactorial decision and this is a single study, this analysis suggests that countries that cannot access Gavi-like prices for ROTARIX® and RotaTeq® may have an economic incentive to consider new products.

There are several limitations to our analysis. First, the surveillance in Mongolia focuses on hospitalized cases only. As a consequence, there is limited local data on non-severe rotavirus disease and we had to rely on uncertain assumptions regarding access to care. The study, therefore, may not capture all non-severe cases and may underestimate the costs related to non-severe visits. Furthermore, because surveillance data were an important input into our incidence calculations, these data are also uncertain. Incidence and access to care data are critical inputs and are explored in the scenario analysis. Second, assessing the cost linked to the immunization program was challenging, as immunization program costs in Mongolia are often integrated into more general cost categories [49]. In addition, the country was in the process of updating its comprehensive multi-year plan at the time of this analysis. This made the assessment of incremental health system costs linked to rotavirus vaccination difficult so we elected to use internationally validated estimates. This approach obliged us to assume a similar incremental health system cost for the delivery of several products. This is a critical consideration when considering alternative products, and we were not able to capture cost differentials linked to specific product characteristics such as volume.

As Mongolia weighs the decision to introduce rotavirus vaccine into its routine childhood immunization schedule, the results of this analysis suggest that a rotavirus vaccination program will avert substantial disease burden and will likely be highly cost-effective in Mongolia.

## Source of funding

This work was supported by the Bill & Melinda Gates Foundation, Seattle, WA [grant number OPP1147721].

## Conflict of interest statement

The authors have no conflicts to declare.

## Disclaimer

The findings and conclusions of this report are those of the authors and do not necessarily represent the official position of the Centers for Disease Control and Prevention (CDC).
